# Left bundle branch area pacing induced dual bundle branches non-reentrant bigeminy and Takotsubo cardiomyopathy: a case report

**DOI:** 10.3389/fphys.2026.1915000

**Published:** 2026-10-07

**Authors:** Song-Yun Chu, Yuxi Li, Li-Tong Qi, Er-Dong Chen, Jing Zhou

**Affiliations:** Department of Cardiology, Peking University First Hospital, Beijing, China

**Keywords:** bundle branch block, case report, left bundle branch area pacing, Takotsubo cardiomyopathy, ventricular bigeminy

## Abstract

A 76-year-old woman with intermittent advanced atrioventricular block from a previous left septal accessory pathway and atrioventricular (AV) nodal slow pathway ablation for supraventricular tachycardia was referred for pacing. Her echocardiogram showed no structural abnormality and preserved left ventricular ejection fraction (LVEF). Left bundle branch area pacing (LBBaP) was achieved successfully, although frequent premature ventricular complexes (PVCs) were observed during the procedure. In the interrogation before discharge, asymptomatic ST-segment elevation on the precordial electrocardiogram (ECG) necessitated an emergent coronary angiography. No significant stenosis or spasm of coronary arteries was found, while left ventriculography revealed apical dyskinesis suggestive of Takotsubo cardiomyopathy. On closer inspection, frequent monomorphic PVCs developed after each pacing beat and formed a regular bigeminy pattern, indicating rapid LBB stimulation, and the facilitated slow conduction along the adjacent bundle branches induced the dual ventricular response. The resultant frequent desynchronized ventricular activation may have contributed to the stress for cardiac decompensation. Beta blocker was titrated to alter the critical time period matched for the repetitive activation pattern and to suppress sympathetic tension. The bigeminy was eliminated ultimately, and repositioning of the lead was avoided. The ventricular wall motion and LVEF recovered within months. This case highlights the importance of close observation of electrocardiographic monitoring after pacing. Despite the potential benefits for future cardiac function and lower pro-arrhythmic effect than routine pacing modalities, LBBaP may still be associated with new-onset arrhythmia and myocardial injury considering the baseline impairment of the electrical or anatomical substrate. Individualized management should be tailored according to specific scenarios.

## Introduction

Conduction system pacing (CSP), in particular left bundle branch area pacing (LBBaP), has increasingly attracted interest as it preserves physiological left ventricular (LV) activation and generally has better pacing and sensing parameters than His-bundle pacing (HBP) (Huang et al., 2017; Wang et al., 2022). At the same time, LBBaP has been shown to be associated with lower arrhythmia burden than biventricular (BiV) pacing (Herweg et al., 2024). Except for the establishment of physiological ventricular activation and effective resynchronization, ventricular pro-arrhythmia by epicardial coronary venous pacing used in conventional Bi-V cardiac resynchronization therapy (CRT) can be avoided during LBBaP (Herweg et al., 2024). However, we report here a rare case in which frequent “premature ventricular contractions” (PVCs) developed after LBBaP, and the so-called PVCs may have become a trigger for the stress of the cardiomyopathy.

## Case description

A 76-year-old woman with intermittent syncope was referred for pacing. She underwent catheter ablation for supraventricular tachycardia 2 years prior. Advanced atrioventricular block (AVB) was recorded in the report from the left septal accessory pathway and atrioventricular (AV) nodal slow pathway ablation and partially resolved by cortisol infusion. At admission, her 12-lead electrocardiogram (ECG) demonstrated 2:1 AV conduction, occasional PVCs following a conducted sinus activation, and paroxysmal complete AVB. Echocardiogram showed no structural abnormality and preserved left ventricular ejection fraction (LVEF). LBBaP was achieved successfully by using a pre-shaped sheath (C315 His, Medtronic, Minneapolis, MN, USA) and a lumenless lead (SelectSecure 3830, Medtronic). Notably, increased PVCs were observed immediately after the lead implantation, mostly showing a regular pacing and PVC bigeminy pattern (**Figure 1**). As seen in the past implantation procedure, we took the PVCs as irritability of the myocardium. As no palpitation or chest pain symptoms were reported and with satisfactory pacing parameter measurements, as well as concerns about there being no better locations for qualified left bundle branch (LBB) capture, we made no further adjustment for the lead position. In the routine ECG before discharge, however, ST-segment elevation was noted in the precordial leads (**Figure 1**).

**Figure 1:**
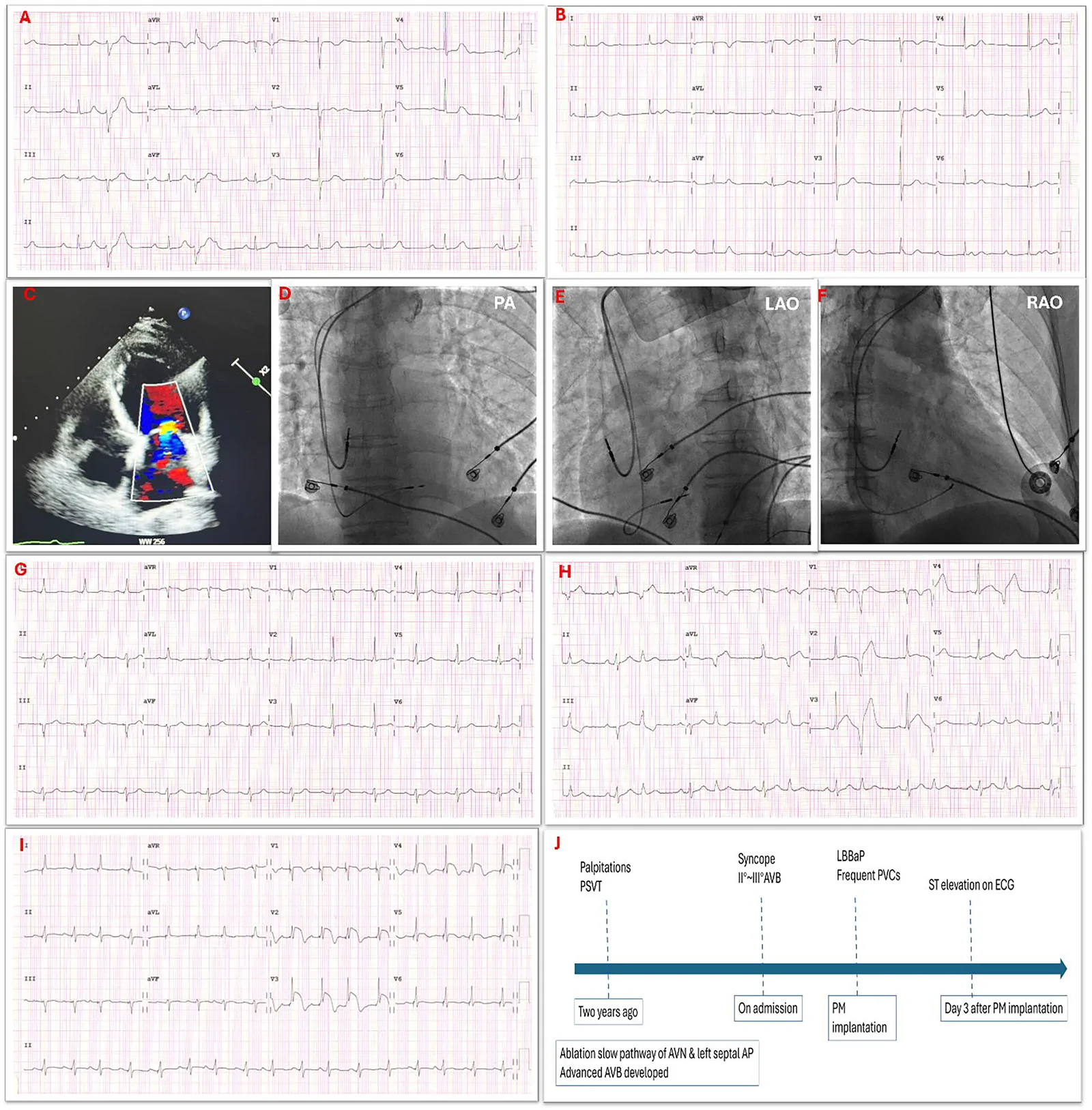
**(A)** Baseline 12-lead electrocardiogram (ECG) showed 2:1 atrioventricular block (AVB). Some sporadic premature ventricular complex (PVC) could be observed after the conducted sinus beats. **(B)** Baseline ECG occasionally showed complete AVB with junctional escape rhythm. **(C)** Baseline normal cardiac geometry, wall motion, and systolic function on echocardiography. **(D–F)** Intraoperative fluoroscopy of the lead positions. **(G)** Left bundle branch area pacing (LBBaP) with bipolar pace setting created a narrow QRS pattern similar to the native ones with a slight leftward electrical axis deviation. **(H)** PVCs after the LBBaP pacing beats showed ventricular bigeminy with inferior axis. **(I)** Routine ECG recording before discharge (day 3 after the LBBaP implantation procedure) suggested ST-segment elevation on precordial leads. **(J)** Timeline of the historical and current information.

## Diagnostic assessment, therapeutic intervention, and follow-up

Although no chest pain symptom could be recalled for this patient, acute coronary syndrome was suspected as widespread ST-elevation in the ECG was shown. Emergent coronary angiography was performed immediately, which showed no significant stenosis or spasm in her coronary arteries or branches, while left ventriculography showed apical dyskinesis and basal hyperkinesis suggestive of typical Takotsubo syndrome (TTS) (**Figure 2**). No evidence of inflammation and a persistent low-level increase of cardiac troponin I with no dynamic change also support the diagnosis.

**Figure 2:**
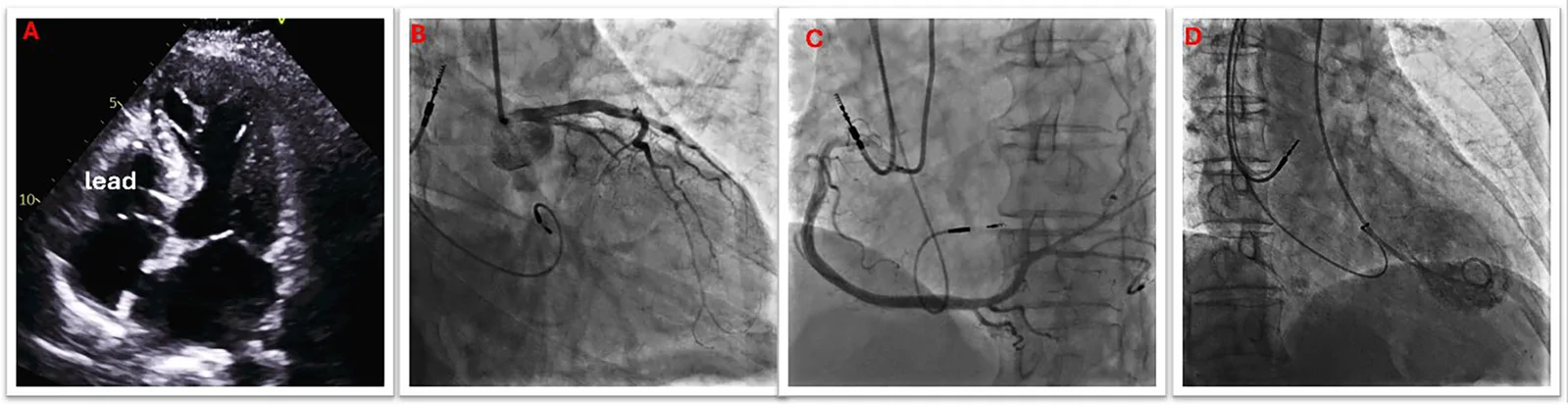
**(A)** Transthoracic echocardiography demonstrated hypokinesis in the anteroseptal and apical myocardium and reduced left ventricular ejection fraction (LVEF). **(B, C)** Neither significant stenosis nor spontaneous vasospasm was found in coronary angiography. **(D)** Left ventriculography showed akinesia in the anteroseptal and apical areas and a hypercontractile base.

TTS is commonly triggered by acute emotional or physical stressors. There are reports of TTS in the perioperative period or after implantation of cardiac implantable electronic devices (Yalta et al., 2026). A high sympathetic tone could drive both TTS and frequent PVCs. At the same time, the high burden of PVCs may become another form of stressor as a superimposed factor that could contribute to the TTS. Notably, PVCs occurring after the LBBaP were monomorphic, with consistent coupling intervals suggestive of the same origin and conduction routes, which is in support of the precursor role of PVCs rather than the consequence of TTS (majority of polymorphic PVCs). Upon review of the ECG and electrogram (EGM) recorded during the procedure, we noticed that the spontaneous PVCs before the lead implantation only occurred after the occasionally conducted native activations, but not during complete AVB. The PVCs after unipolar pacing beats showed a similar QRS morphology of the LBBB pattern to those of the spontaneous PVCs after the conducted sinus beats. The phenomenon suggests that the rapid activation of proximal LBB facilitated nearby impaired but slow conduction of His-RBB (right bundle branch), resulting in a dual activation of ventricles resembling to bigeminies. Bipolar pacing may have altered the exit of the slow activation and produced a similar precordial QRS pattern, but with a different frontal axis of the PVCs. Bipolar pacing may have also directly stimulated the RBB, resulting in a slightly earlier conduction through the RBB compared with unipolar pacing (**Figure 3**).

**Figure 3:**
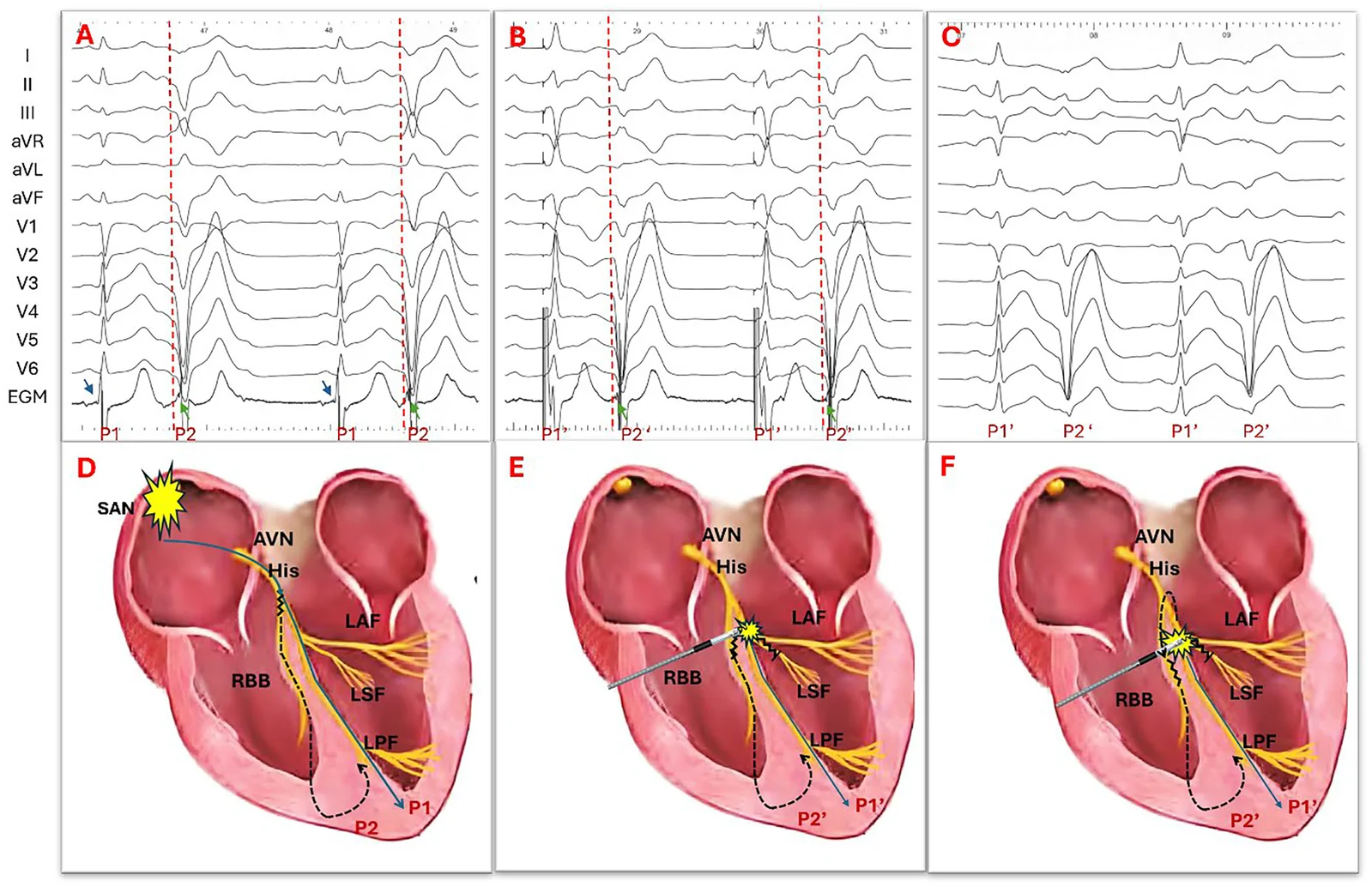
**(A)** Sporadic native conducted sinus rhythm followed by the premature ventricular complex (PVC) with left bundle branch block (LBBB) pattern, negative concordance in the precordial leads, and superior frontal axis suggesting an exit of the right ventricular apex. The PVCs were never observed in time of complete atrioventricular conduction block and junctional rhythms. Blue arrows denote the recorded antegrade left bundle branch (LBB) potentials. **(B)** Pacing and PVC beats bigeminy was observed after left bundle branch area pacing (LBBaP). Please note the similar morphology of PVCs to that in **(A)** and the fixed coupling intervals between the pacing beats and the PVCs. In the electrogram (EGM) of the LBB channel, a late local potential (in comparison to the start of the QRS) was recorded (green arrows) in correspondence to the PVC, which is in support of the conduction from the distal to the proximal septum (retrograde LBB activation). These observations suggest that the slow conduction facilitated by normal atrioventricular (AV) nodal conduction was blocked in the LBB, but inserted into the right bundle branch and activated the apical ventricle after the previous normal conducted beats, similar to the mechanism of a nodofascicular pathway activation. LBBaP led to the repeated recruitment of the slow conduction of the perinodal region and the dual bundle branch non-reentrant ventricular activation. **(C)** LBBaP with bipolar pacing eliminated the R′ wave in lead V1 and resulted in a narrow QRS complex similar to the native ones. The bigeminy was also evident, but with the PVCs showing an opposite direction at the inferior limb leads, suggesting higher exits at the frontal axis. Please note the same pacing and PVC morphology in this intraoperative recording and the electrocardiogram (ECG) after the implantation of the pacemaker, as shown in **Figure 1H**. (**D**–**F**) Schematic diagram of the putative mechanism underlying the arrhythmias.

Another form of bigeminy with RBB-like PVCs provided evidence of an alternative dual activation introduced by LBBaP and facilitated slow conduction along the left anterior fascicular fiber (**Figure 4**).

**Figure 4:**
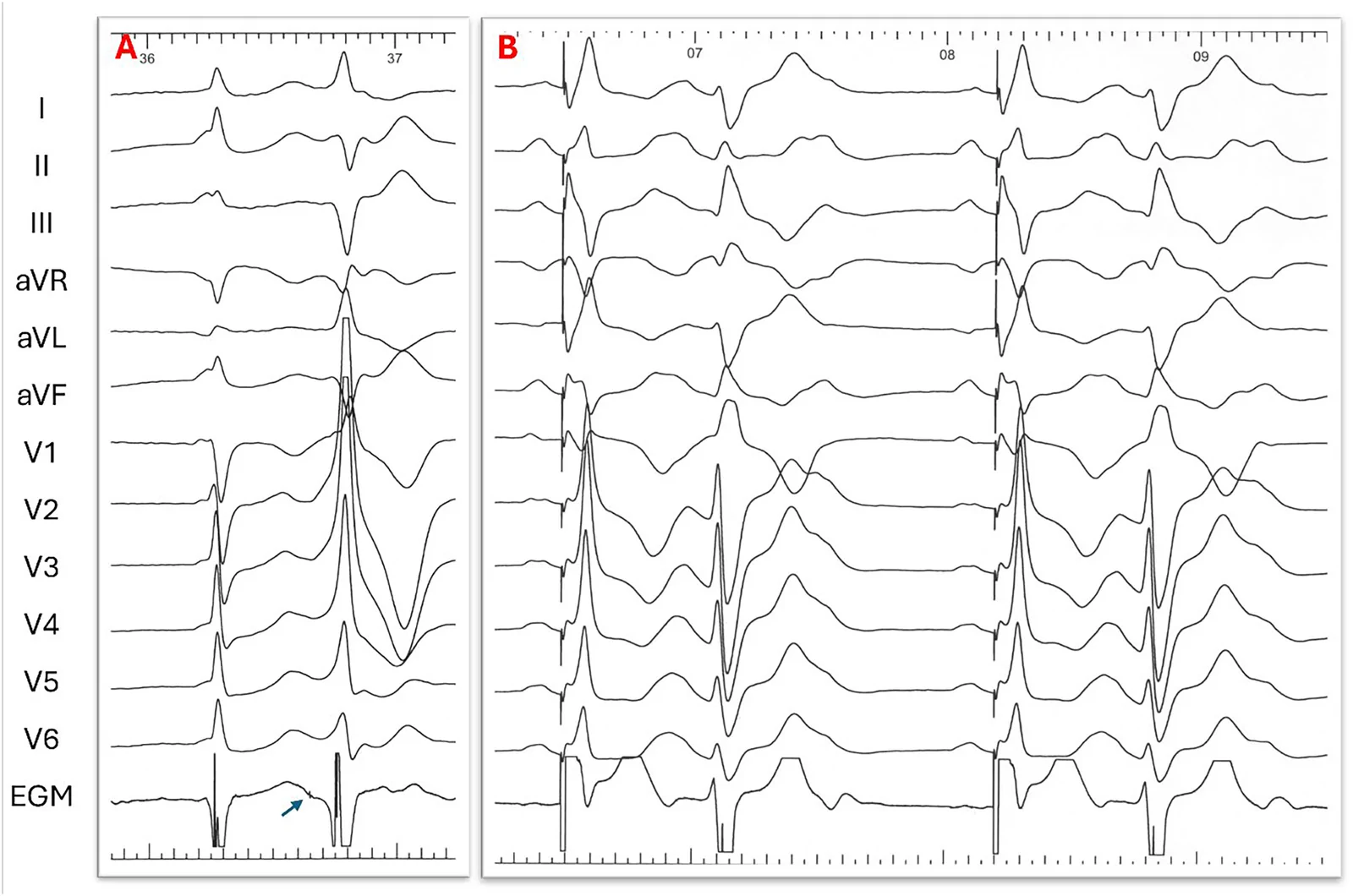
**(A)** Spontaneous premature ventricular complex (PVC) with the morphology of left septal branch block (LSPB) occurred after a local potential (blue arrow) recorded in the electrogram (EGM), suggesting scar border origin from the left septum that might have resulted from previous ablation. Please note that the left bundle branch area pacing (LBBaP) led to a right bundle branch block and an LSPB pattern (prominent anterior QRS forces in the *horizontal plane*), also suggesting the slow conduction area region near the left septum. **(B)** Another form of pacing and PVC bigeminy. LBBaP pacing QRS followed by PVC with the pattern of left posterior fiber block (LPFB). The PVCs were not observed in complete AV block and junctional rhythms. We suspect that this is another form of repetitive activation of dual bundle branches.

Prior ablation-induced injury in multiple regions of the conduction system may have created a vulnerable environment for the multiple delays in the impulse conduction. Alternating bundle branch or fascicle activation with dual ventricular response and resultant frequent desynchronized ventricular activation may have contributed to the stress that induced cardiac dysfunction. The beta blocker was titrated to alter the critical time period matched for the repetitive activation pattern and to suppress sympathetic tension. The bigeminy was eliminated ultimately, and repositioning of the lead was avoided. The ventricular wall motion and cardiac function recovered within 4 months.

## Discussion

We presented a rare case of dual bundle branch alternative activation-associated bigeminy and Takotsubo cardiomyopathy that occurred after a successful LBBaP procedure.

The patient was admitted for irreversible AV conduction block and symptomatic bradycardia. With the expected high percentage of ventricular pacing, physiological LBBaP was the preferred pacing modality for the potential protection of acute hemodynamic response, long-term synchronized and preserved LV function comparable to BiV pacing (Liang et al., 2022; Wang et al., 2022). Successful capture of the LBB conduction system was demonstrated by typical RBBB morphology, a short and fixed peak time for the V6 R wave, and the LBB potentials in the EGM. However, the unexpected ST-elevation changes and the development of cardiac dysfunction were puzzling.

Although a unique risk of injury to the septal perforator arteries for LBBaP has been suggested, the absence of chest pain symptoms throughout the course made the possibility minimal. Diagnostic coronary angiography provided a direct denial demonstration. On the other hand, the frequent PVCs after the implantation raised concerns for PVC-induced cardiomyopathy presenting as an apical ballooning syndrome. In fact, cardiomyopathy induced by PVCs is mechanistically different from a tachycardia-mediated cardiomyopathy produced by regular ventricular tachy-pacing, with more severe LV systolic dysfunction due to the irregularity and dyssynchrony of the ventricles (Walters et al., 2018). The severity of the hemodynamic derangement associated with ectopic beats exhibits a dose effect on cardiomyopathy development (Walters et al., 2018). Although the majority of reports described cardiac dysfunction developing after a long time, continued PVC bigeminy after the procedure may have contributed to the rapid development of cardiac dysfunction.

On the other hand, we noted the significantly increased PVCs shortly after the device implantation that appeared to be triggered by pacing. Pacing-related pro-arrhythmia has been reported with conventional right ventricular (RV) pacing and LV epicardium (coronary sinus) pacing (Shukla et al., 2005; Lee et al., 2009; Hayase et al., 2018). As the I-CLAS study showed that LBBAP led to fewer atrial and ventricular tachyarrhythmias than BiV pacing (Herweg et al., 2024), LBBAP-associated pro-arrhythmia has been increasingly reported in recent literature. One case described an electrical storm from monomorphic or pleomorphic ventricular tachycardia (VT) months after LBBaP, with the triggered PVC morphology suggestive of an origin from the proximal interventricular septum. The resolvent of the VTs required replacement of the LBBaP lead to routine RV endocardial pacing, upgrading to an implantable cardioverter defibrillator (ICD) device, and strengthened medication control (Bhatt et al., 2024). Another case reported multiple episodes of VTs after LBBaP, attributed to the pacing stimulation and which necessitated removal of the LBBaP lead, upgrading of the device to CRT defibrillator (CRT-D), and ablation (Tanawuttiwat et al., 2023). Both of the cases had structural heart disease, and LBBaP led to PVCs as triggers to initiate the VTs. Vijayaraman et al. presented an intra-procedural bundle branch reentry VT at the time of LBBaP lead implantation and eliminated it by BiV pacing afterwards (Ponnusamy and Vijayaraman, 2021). The patient had cardiomyopathy and reduced LVEF. Recently, sustained bundle branch reentrant VT with two different morphologies was reported 1 month after LBBaP implantation in a patient without apparent structural heart disease. Repeated ablation at the lead location and repositioning of the lead to the RV septum comprised the final intervention for the VT (Zhang et al., 2026). Prior postulated mechanisms of pro-arrhythmia of pacing may be local irritability (Germano et al., 2006), which is supported by a relatively higher incidence of VT occurring early after lead implantation. On the other hand, local fibrosis or scars provided a substrate for slow conduction and unidirectional conduction block and may lead to reentry. Pacing close to the critical region of slow conduction may have a pro-arrhythmic effect, known as pace-mapped induction of VT (Tung et al., 2012).

Our presented case is in line with prior cases showing ventricular arrhythmia shortly after LBBaP, and the resultant PVCs appeared to be associated with the His–Purkinje system. However, our case is different from prior cases in that the so-called PVCs were not the direct consequence of LBBaP pacing, but facilitated slow conduction from the adjacent lesioned component of the conduction system by the rapid activation of LBB in an apparent structural normal heart. The dual bundle branch alternative activation was mechanically similar to the dual AV nodal non-reentrant tachycardias that happened in patients with AV nodal fast and slow pathway. In our case, the two different alternative activation modes of branches or fascicles suggested multiple impairments in the proximal conduction systems, which are assumed to be the complications of prior ablation. Notably, spontaneous “PVCs” before the pacemaker implantation only occurred after an occasional native conducted impulse, which also supports the pro-arrhythmic effect by stimulating the critical region of slow conduction. When we achieved the LBBaP successfully, each pacing stimulation acted as an initiation of dual fast and slow activation and created ventricular bigeminies. A significantly increased “PVC” burden may become the culprit for the exacerbation of cardiac dysfunction.

Titrated beta blockers and possible remodeling progress after the TTS may have changed the critical conditions for dual activations. From the patient’s perspective, she was fortunate because the arrhythmia was observed to have decreased and revision of the LBBaP lead was avoided.

Despite the favorable evolution of the course in our case, potential pro-arrhythmic risks of LBBaP remain a concern for future adverse outcomes. Considering that more patients with structural heart disease or significant conduction lesions may receive LBBaP as an important physiological pacing modality, our case stresses the need for careful observation of suspected new-onset arrhythmia during implantation and follow-up. For patients with a high burden of pacing-associated arrhythmia, the potential impact on cardiac function should be weighed against the benefits of CSP. Treatment strategies must be individualized for each patient.

## Data Availability

The original contributions presented in the study are included in the article/supplementary material. Further inquiries can be directed to the corresponding author.
